# Palatal tremor after lithium and carbamazepine use: a case report

**DOI:** 10.1186/1752-1947-4-176

**Published:** 2010-06-11

**Authors:** Rajnarayan Mahasuar, Anju Kuruvilla, KS Jacob

**Affiliations:** 1 Department of Psychiatry, Christian Medical College, Vellore 632002, India

## Abstract

**Introduction:**

Palatal tremor, characterized by rhythmic contractions of the soft palate, can occur secondary to pathology in the dentato-rubro-olivary pathway, or in the absence of such structural lesions. Its pathogenesis is only partially understood. We describe a case of probable drug-induced palatal tremor.

**Case presentation:**

A 27-year-old Indian man had taken carbamazepine and lithium for 7 years for the treatment of a manic episode. He presented with a one-year history of bilateral rhythmic oscillations of his soft palate and tremors of his tongue. There were no other abnormalities detected from his examination or after detailed investigation.

**Conclusion:**

Palatal tremors may result from medication used in the treatment of psychiatric disorders.

## Introduction

Palatal tremor (PT), previously referred to as palatal myoclonus, is characterized by rhythmic contractions of the soft palate. Symptomatic PT is diagnosed secondary to lesions in the dentato-rubro-olivary pathway. On the other hand, essential PT has no known structural lesions.

Bipolar affective disorder is a mental illness with an annual incidence of less than 1% and a lifetime prevalence of 0% to 7.8% [1]. It is characterized by recurrent periods of depression that alternate with periods of hypomania or mania. Mood stabilizers, such as lithium, sodium valproate and carbamazepine, are recommended in the treatment of acute mania. These are also used as prophylactic agents in the treatment of bipolar disorders [2]. We report the case of a patient with PT associated with the prolonged use of lithium and carbamazepine.

## Case presentation

A 27-year-old Indian man presented with complaints of a clicking sound upon partially opening his mouth. He had experienced this symptom for the past year.

Relatives had observed that the sounds were absent when he was asleep. Our patient had had a single episode which was suggestive of mania with psychotic symptoms seven years prior to presentation. Since then he had been taking a combination of lithium and carbamazepine continuously as he felt there had been mild fluctuations in his mood when he had attempted to discontinue the drugs. There had been no changes in his drug dosage prior to the onset of his palatal problems. He had no history of using antipsychotic medication. He also had no other significant personal, medical or family history of neuropsychiatric illness. On his detailed psychiatric evaluation, no anxiety, mood or psychotic symptoms were found.

The results of our patient's physical examination revealed bilateral rhythmic oscillations of his soft palate associated with a clicking sound and tremors of the tongue,which were not synchronous with his palatal movements. The clicking sound was not audible when his mouth was closed or wide open. With distraction, his tongue tremors reduced in intensity but no obvious change in the clicking sound was noticed. As such, there was no obvious evidence that our patient's palatal movements were under voluntary control. He had no difficulty in speaking or swallowing. There were also no abnormalities in his eye movements.

An examination of his other systems did not reveal any abnormalities. The results of his routine blood tests, as well as liver, kidney and thyroid function tests, were all normal. The results of his brain magnetic resonance imaging (MRI), electroencephalography (EEG), electromyography (EMG), and a laboratory work-up for Wilson's disease were also normal. His serum carbamazepine and lithium levels were 5.47 mcg/mL (target blood level 4 mcg/mL to 12 mcg/mL) and 0.57 mmol/L (target blood level 0.8 mmol/L to 1.2 mmol/L), respectively.

We considered a diagnosis of tardive PT, so his lithium dosage was tapered off and then stopped. We continued his carbamazepine dosage and started him on a therapy of clonazepam at 0.25 mg twice daily. Subsequently, his visible tongue tremors were reduced in a week and by the end of one month he subjectively reported an improvement of about 60% in his tremors and clicking sound. Over the next three months of follow-up, our patient reported a periodic fluctuation in the intensity of his symptoms but with no further sustained improvement.

## Discussion

Palatal tremor is characterized by rhythmic movements of the soft palate. These movements are repetitive audible clicks [3] which are thought to be related to the opening and closing of the eustachian tube [4]. In the past, several terms have been used to describe this condition. At present, however, it is thought to be best classified phenomenologically as a tremor [5].

Symptomatic PT is associated with hypertrophic olivary degeneration that is visible on an MRI scan. It also has multiple causes, the most common being vascular. This particular PT persists during sleep and is associated with ocular and cerebellar signs.

Meanwhile, essential PT is thought to be heterogeneous and modified by sleep, neck position or mouth opening, and rarely involves the eyes [6]. It is proposed that some 'essential' tremors of the palate may in fact be centrally generated [6,7] while others may be peripheral or mechanical in origin, psychogenic, voluntary, or multifactorial in nature [5]. A psychogenic origin is suspected when the movement is reduced by distraction, when other psychopathology is present, when the symptoms respond spontaneously to a placebo, or improvement occurs faster than expected by therapy [8]. Epileptic palatal myoclonus is uncommon and is associated with cortical dysfunction on EEG [9].

In our patient, in the absence of other causes, the possibility of a tardive, drug-induced PT was considered. Lithium, in therapeutic and toxic levels, is known to produce a variety of movement disorders. A low-amplitude and fast postural tremor of the hands, which can worsen during activities requiring fine motor control, is commonly observed [10]. Myoclonus of the limbs [11] and tardive syndromes [12] have also been reported. Although carbamazepine can produce tremors, especially when combined with lithium and neuroleptics, and can worsen essential tremors [13], it is also recommended as a treatment of PT [14] and myoclonus [15]. While drug-induced PT has been observed with the use of ciprofloxacin [16], to the best of our knowledge this is the first report of palatal and lingual tremors associated with the use of lithium and carbamazepine.

Since the withdrawal of lithium and the introduction of clonazepam occurred simultaneously in our case, we cannot assume that the reduction in symptoms was solely due to the removal of lithium. The persistence of the symptoms despite the complete withdrawal of lithium may suggest that carbamazepine also has a role in the manifestation of his symptoms.

The pharmacological treatment for PT includes a variety of agents such as anticonvulsants, benzodiazepines, anticholinergic agents, calcium channel blockers (flunarizine), 5-hydroxytryptamine (5HT) and 5HT agonists (sumatriptan), nootropics (piracetam), placebos and botulinum toxin [17]. Tonsillectomy and other local therapies [7], relaxation techniques, voluntary mechanisms [17] such as the Valsalva maneuver, and dental devices [18] have also been attempted. However, the response of patients to these interventions is varied.

## Conclusion

Palatal tremor is an uncommon movement disorder that can occur following the prolonged use of psychotropic medication. Clinicians prescribing these drugs must take measures to reduce the risk of such disorders by carefully monitoring for the development of associated symptoms in their patients.

## Consent

Written informed consent to publish could not be obtained despite reasonable attempts. All efforts have been made to protect the identity of the patient and there is no reason to believe that he would object to its publication.

## Competing interests

The authors declare that they have no competing interests.

## Authors' contributions

RM and KSJ took care of our patient and contributed to the conception of the case report. AK undertook the literature review and drafted the manuscript. All authors read and approved the final manuscript.
